# A Framework for Assessing High School Students' Statistical Reasoning

**DOI:** 10.1371/journal.pone.0163846

**Published:** 2016-11-03

**Authors:** Shiau Wei Chan, Zaleha Ismail, Bambang Sumintono

**Affiliations:** 1 Department of Production and Operation Management, Faculty of Technology Management and Business, Universiti Tun Hussein Onn Malaysia, Batu Pahat, Johor, Malaysia; 2 Department of Educational Sciences, Mathematics and Creative Multimedia, Faculty of Education, Universiti Teknologi Malaysia, Skudai, Johor, Malaysia; 3 Institute of Educational Leadership, University of Malaya, Kuala Lumpur, Malaysia; Center for BrainHealth, University of Texas at Dallas, UNITED STATES

## Abstract

Based on a synthesis of literature, earlier studies, analyses and observations on high school students, this study developed an initial framework for assessing students’ statistical reasoning about descriptive statistics. Framework descriptors were established across five levels of statistical reasoning and four key constructs. The former consisted of idiosyncratic reasoning, verbal reasoning, transitional reasoning, procedural reasoning, and integrated process reasoning. The latter include describing data, organizing and reducing data, representing data, and analyzing and interpreting data. In contrast to earlier studies, this initial framework formulated a complete and coherent statistical reasoning framework. A statistical reasoning assessment tool was then constructed from this initial framework. The tool was administered to 10 tenth-grade students in a task-based interview. The initial framework was refined, and the statistical reasoning assessment tool was revised. The ten students then participated in the second task-based interview, and the data obtained were used to validate the framework. The findings showed that the students’ statistical reasoning levels were consistent across the four constructs, and this result confirmed the framework’s cohesion. Developed to contribute to statistics education, this newly developed statistical reasoning framework provides a guide for planning learning goals and designing instruction and assessments.

## Introduction

Today, statistical reasoning has become a ubiquitous part of many disciplines, such as business [1], education, and engineering. Nevertheless, high school students appear to have poor statistical reasoning ability [2], and they often harbor numerous misconceptions about statistical reasoning, as found in our preliminary and earlier studies. For instance, in descriptive statistics, students frequently make mistakes in answering questions regarding measures of central tendency [3,4] and variability [5–7]. Statistical reasoning is a neglected area, particularly compared to the areas of statistical literacy and statistical thinking [8]. In addition, statistical reasoning is not adequately covered in Malaysian mathematics textbooks. Statistical reasoning must be incorporated into the Malaysian curriculum to foster students’ conceptual understanding of statistical concepts. Hence, this study aims to bridge these gaps by focusing on statistical reasoning about descriptive statistics. In this context, statistical reasoning is defined as ‘the way people reason with statistical ideas and make sense of statistical information. It involves making interpretations based on sets of data or statistical summaries of data, where students need to combine ideas about data and chance to make inferences and interpret statistical results’ [9] (p.101).

The initial statistical reasoning framework in the present study was developed from the five levels of statistical reasoning of Garfield’s [10] model and the four key constructs of Jones et al.’s [11] framework [12]. The development and initial validation of the statistical reasoning assessment tool based on the aforementioned initial statistical reasoning framework were conducted by Chan and Ismail [12,13]. The initial framework and assessment tool from Chan and Ismail [12] were employed in the current study. Few studies to date have focused on the five levels of statistical reasoning, especially in secondary students. More studies have focused on the four constructs, but research on the integration of information technology with the four processes remains scarce. Furthermore, although some studies have investigated reasoning related to measures of central tendency, variability and distribution, this domain is far from a complete and coherent statistical reasoning framework. This area is thus addressed in the present study, and in contrast to preceding frameworks, this newly developed framework is more unique and distinctive in combining Garfield’s [10] model with Jones et al.’s [11] framework. As such, the purposes of this study are as follows:

1. To develop an initial framework for assessing students’ statistical reasoning;
2. To construct a tool for assessing students’ statistical reasoning; and
3. To refine and validate the initial framework for assessing students’ statistical reasoning.

## Theoretical Considerations

### Five levels of Statistical Reasoning

The model of statistical reasoning proposed by Garfield [10] has five levels: idiosyncratic reasoning (Level 1), verbal reasoning (Level 2), transitional reasoning (Level 3), procedural reasoning (Level 4), and integrated process reasoning (Level 5). At Level 1, students can use several statistical words and symbols but cannot completely comprehend and relate them to the appropriate information. Thus, their answers are often inaccurate. For example, such students might know the term ‘standard deviation’ but are unable to use it correctly. Students at Level 2 can perform better because they know the definitions of some statistical ideas, but they still fail to apply them correctly. For instance, these students might be able to choose the correct definition of interquartile range, but they cannot answer conceptual questions. At Level 3, students are capable of recognizing one or two aspects of the statistical process, but they cannot practically incorporate these concepts to find answers. For example, such students can distinguish the shape, measures of central tendency and variability of graphical representations but cannot integrate them into their solutions. At Level 4, students can identify statistical processes accurately, but they still lack the ability to fully comprehend or integrate them. For example, these students might be able to recognize the concept of averages but unable to fully interpret it. Students at the highest level, which is Level 5, have full knowledge of statistical processes and are competent in coordinating rules and behavior as well as elucidating the process in their own words [10].

The model introduced by Biggs and Collis [14,15], known as the Structure of Observed Learning Outcomes (SOLO) taxonomy, was established on a theoretical basis [16] that has been widely discussed in cognitive models of statistical reasoning development. Jones et al. [11] and Mooney [16] found that many students’ statistical thinking levels lie within the four cognitive thinking levels of the SOLO model. Thus, to formulate our new framework of statistical reasoning, we assumed that the statistical reasoning levels of students across the four constructs can also be described using the SOLO model. This model has five modes of functioning [15]: sensorimotor (from birth), ikonic (from approximately 18 months), concrete-symbolic (from approximately 6 years), formal (from approximately 14 years), and post-formal (from approximately 20 years). As noted by Panizzon, Pegg and McGee [17], students who act in response to their physical environment are at the sensorimotor stage, while students who can internalize action in terms of figures are at the ikonic stage. Students who are at the concrete symbolic stage prefer to use symbolic systems such as number schemes, maps, and written words. Students who can also apply abstract ideas are in the formal mode, and students who are skilled in analyzing the essential structure of theories and disciplines are said to be in the post-formal mode.

The SOLO model also has five levels of understanding, which are prestructural, unistructural, multistructural, relational, and extended abstract [14]. At the prestructural level, students are perplexed and thus can give only irrelevant responses when solving a task. In short, these students can show only small pieces of evidence related to their learning [18]. At the unistructural level, students can emphasize one related feature of the task and can use some terms correctly as well [18]. As they improve, these students can bring in several task-related features without integrating them, which means that they have reached the multistructural level. Biggs and Tang [18] used the following analogy to describe students at this level: they can see the tree but not the wood because they do not yet fully comprehend the situation. At the relational level, students can combine different features of the task into a coherent whole, and at the extended abstract level, students are capable of generalizing and conceptualizing the incorporated whole to a higher level of abstraction.

In this study, we hypothesized that students at Level 1 (idiosyncratic reasoning) would exhibit elements of the prestructural level in the ikonic mode. Meanwhile, students at Level 2 (verbal reasoning) would display elements of the unistructural level in the concrete symbolic mode. Similarly, elements of the multistructural level in the concrete symbolic mode would be demonstrated by students at Level 3 (transitional reasoning). We also postulated that students at Level 4 (procedural reasoning) would show elements of the relational level in the concrete symbolic mode. Finally, students who reveal elements of the extended abstract level in the formal mode are regarded as having reached Level 5 (integrated process reasoning).

### The Four Constructs

Jones et al. [11] and Mooney [16] mentioned four constructs in their studies: describing data, organizing and reducing data, representing data, and analyzing and interpreting data. These four constructs were adopted from the work of Shaughnessy, Garfield and Greer [19]. Describing data entails directly reading the data revealed in charts, tables, and other graphical displays [16]. Organizing and reducing data involve classifying, organizing, or combining data into synopsis form [16], as well as reducing data using measures of central tendency and variability [11]. Representing data is defined as showing data in graphical form [16]. Analyzing and interpreting data involve recognizing trends and making predictions or inferences from a graphical display [16]. Previous studies have demonstrated that many students often encounter difficulties with these four constructs.

With regard to describing data, many students have found it difficult to read different types of graphs, such as bar graphs and histograms, rendering them incapable of performing various forms of data analysis [20]. Students also tend to see the data as individual entities rather than as a cluster of data [21,22]. Hence, to overcome this tendency, we recommended three sub-processes for describing data in this study: (i) extracting and generating information from the data or graph, (ii) showing awareness of the display attributes of the graphical representation, and (iii) recognizing the general features of the graphical representation. The first sub-process is important in enabling students to obtain explicit information from the data or graphs. The second sub-process is identical to the first sub-process of describing data in Mooney’s [16] study, where students should have an awareness of the display attributes of the graphical representation. In addition, the third sub-process is added to the framework because it is imperative to guide students to see the three ideas (shape, measures of central tendency, and variability) as a whole unit [23] when they recognize the common characteristics of the graphical representations.

For organizing and reducing data, earlier studies revealed that many students have misconceptions about measures of central tendency, including the mean [24–27], median [27], mode [28], and measures of variability [29–31]. Sharma [32] claimed that students may be bewildered when both measures are combined in one task. Therefore, three sub-processes of organizing and reducing data were proposed in this study to address this issue: (i) organizing data into a computer system; (ii) reducing data using measures of central tendency, either by calculation or aided by technology; and (iii) reducing data using measures of spread, either by calculation or aided by technology. These three sub-processes are different from those used in earlier studies because they involve the utilization of information technology, an element that was not previously emphasized. For the first sub-process, students were required to organize the data in the computer system rather than manually. Furthermore, the students were asked to use measures of central tendency and variability to reduce their data manually and to use computerized computation in the second and third sub-processes. After they had performed these calculations manually, the students were required to check their answers using computers.

Representing data using different mathematical graphics, such as histograms [27,33,34], box plots [35], and bar graphs [36], can be a daunting task for many students. Consequently, we suggest three sub-processes of representing data in this study: (i) demonstrating data sets graphically using a computer, (ii) identifying different representations for the same data set, and (iii) judging the effectiveness of two different representations for the same data. Admittedly, these three sub-processes also involve the utilization of information technology. In the first sub-process, students were to present their data by dragging the figure dynamically and drawing different graphical representations using GeoGebra software. The second sub-process is similar to the second sub-process of describing data in Mooney’s [16] study, in which students were required to identify different graphical representations for the same set of data. Moreover, the third sub-process is similar to the third sub-process of describing data in Mooney’s [16] study, as it asks students to assess the efficacy of two different representations for the same data. In contrast to those in previous studies, these three sub-processes involve not only evaluating the process of creating graphs but also making sense of the graphs in order to develop more sophisticated reasoning in representing data [37].

With reference to analyzing and interpreting data, many students face difficulties in comparing groups or distributions, as shown by previous studies such as those of Pfannkuch and Reading [38] and Ciancetta [39]. Thus, to overcome this tendency, this study recommends three sub-processes of analyzing and interpreting data: (i) making comparisons within the same data set; (ii) making comparisons between two different data sets; and (iii) making a prediction, inference or conclusion among the data or graphs. The first sub-process requires students to make comparisons within the same set of data, while the second requires them to make comparisons between two different sets of data. The first and second sub-processes correspond to the first and second sub-processes in Mooney’s [16] study. For the third sub-process, students were asked to make a prediction, inference or conclusion from data or graphs. The process of making a prediction is equivalent to the second sub-process in Jones et al.’s [11] study. Meanwhile, the process of making an inference is comparable to the third sub-process in Mooney’s [16] study. Although the process of drawing a conclusion was not included in earlier studies, it is crucial for students to know how to draw conclusions from the data or graphs at the end of a task.

These four constructs, together with the aforementioned five levels of statistical reasoning, constitute the initial statistical reasoning framework of this study, as displayed in Table 1.

**Table 1:** Initial Statistical Reasoning Framework.

Construct	Level	Level 1 Idiosyncratic	Level 2 Verbal	Level 3 Transitional	Level 4 Procedural	Level 5 Integrated Process
Describing Data		D1L1	D1L2	D1L3	D1L4	D1L5
		Does not extract and generate idiosyncratic or relevant information from the data or graph	Extracts and generates some information from the data or graph verbally, but interpretation is ambiguous or unclear	Extracts and generates one or two dimensions of information from the data or graph	Extracts and generates information from the data or graph correctly	Extracts and generates information from the data or graph completely
		D2L1	D2L2	D2L3	D2L4	D2L5
		Does not show awareness of the displayed attributes of graphical representation	Shows awareness of the displayed attributes of graphical representation orally but is only partly correct	Shows little awareness of the displayed attributes of graphical representation	Shows some awareness of the displayed attributes of graphical representation	Shows complete awareness of the displayed attributes of graphical representation
		D3L1	D3L2	D3L3	D3L4	D3L5
		Does not recognize general features of the graphical representation	Recognizes general features of the graphical representation in words but is only partly accurate	Recognizes one or two general features of the graphical representation	Recognizes general features of the graphical representation accurately	Recognizes general features of the graphical representation completely
Organizing and Reducing Data		O1L1	O1L2	O1L3	O1L4	O1L5
		Unable to organize the data into a computer system	Provides oral statements when organizing the data into a computer system but is only partly correct	Organizes the data into a computer system with major mistakes	Organizes the data into a computer system with minor mistakes	Organizes the data into a computer system correctly
		O2L1	O2L2	O2L3	O2L4	O2L5
		Unable to reduce the data using measures of central tendency, either by calculation or aided by technology	Reduces the data using measures of central tendency in words, either by calculation or aided by technology, but is accurate only to some extent	Reduces the data using measures of central tendency with major errors, either by calculation or aided by technology	Reduces the data using measures of central tendency with minor errors, either by calculation or aided by technology	Reduces the data using measures of central tendency completely, either by calculation or aided by technology
		O3L1	O3L2	O3L3	O3L4	O3L5
		Unable to reduce the data using measures of spread, either by calculation or aided by technology	Reduces the data using measures of spread orally, either by calculation or aided by technology, but is accurate only to some extent	Reduces the data using measures of spread with major faults, either by calculation or aided by technology	Reduces the data using measures of spread with minor faults, either by calculation or aided by technology	Reduces the data using measures of spread completely, either by calculation or aided by technology
Representing Data		R1L1	R1L2	R1L3	R1L4	R1L5
		Demonstrates data sets graphically using the computer without precise display	Provides verbal statements when demonstrating data sets graphically using the computer but is only partially correct	Demonstrates data sets graphically using the computer with major errors	Demonstrates data sets graphically using the computer with minor errors	Demonstrates data sets graphically using the computer with a valid display
		R2L1	R2L2	R2L3	R2L4	R2L5
		Does not identify different representations for the same data set	Identifies different representations for the same data set in words but is only partially accurate	Identifies one or two aspects of different representations for the same data set	Identifies different representations for the same data set correctly	Identifies different representations for the same data set in a complete and comprehensive way
		R3L1	R3L2	R3L3	R3L4	R3L5
		Does not judge the effectiveness of two different representations for the same data set	Judges the effectiveness of two different representations for the same data set orally but is only partially correct	Judges one or two elements of the effectiveness of two different representations for the same data set	Judges the effectiveness of two different representations for the same data set accurately	Judges the effectiveness of two different representations for the same data set completely
Analyzing and Interpreting Data		A1L1	A1L2	A1L3	A1L4	A1L5
		Does not make comparisons within the same data sets	Makes some comparisons within the same data sets verbally, but comparisons are incomplete	Makes one or two comparisons within the same data sets	Makes comparisons within the same data sets correctly	Makes comparisons within the same data sets completely
		A2L1	A2L2	A2L3	A2L4	A2L5
		Does not make comparisons between two different data sets	Makes comparisons between two different data sets in words, but comparisons are somewhat incorrect	Makes one or two comparisons between two different data set	Makes comparisons between two different data sets accurately	Makes comparisons between two different data sets completely
		A3L1	A3L2	A3L3	A3L4	A3L5
		Does not make predictions, inferences or conclusions from the data or graphs	Makes predictions, inferences or conclusions from the data or graphs in words, but these are incomplete	Makes one or two predictions, inferences or conclusions from the data or graphs	Makes predictions, inferences or conclusions from the data or graphs in an appropriate way	Makes predictions, inferences or conclusions from the data or graphs in a complete and comprehensive way

## Methodology

### Validation Process

As noted above, an initial statistical reasoning framework was formulated from the five statistical reasoning levels and four constructs. A statistical reasoning assessment tool was then created based upon this initial framework, which was employed during the task-based interview [12]. Subsequently, the responses given by the students were analyzed, and the initial framework was refined accordingly. However, the characteristics of statistical reasoning cannot be determined from the responses, given the inappropriate statistical reasoning assessment tool items. For instance, in response to one of the initial items for describing data, ‘What are the highest and lowest amounts of proteins (in grams) for various fast food sandwiches?’, the students simply stated the highest and lowest values; therefore, their actual statistical reasoning could not be assessed. Therefore, the phrase ‘explain your answer’ was added to that question to elicit more information regarding their statistical reasoning. In addition, the earlier refinement of the framework was inadequate because the descriptors for Levels 2 and 3 were similar, making it difficult to clearly determine the students’ statistical reasoning levels. Some of the descriptors also did not closely reflect the definitions of the five levels of statistical reasoning. For example, the initial descriptors of Level 2 and 3 for organizing and reducing data were ‘provides oral statements when organizing the data into a computer system but is only partly correct’ and ‘organizes the data into a computer system with major mistakes’, respectively. Determining the students’ responses under these two descriptors was difficult because partially correct answers were somewhat similar to major mistakes. To match the definition of verbal reasoning, the descriptor of Level 3 was changed to ‘unable to relate to the actual data or graph’. Hence, both the framework and the statistical reasoning assessment tool were revised. The task-based interview was performed a second time using the revised assessment tool, and the statistical reasoning levels of the students were re-examined based on the refined framework. The appropriateness of the descriptors in assessing the statistical reasoning levels validated the statistical reasoning framework. The process of validating the framework was modified from earlier studies [11,16,40–43] and can be described as follows:

1. Construct the statistical reasoning assessment tool based on the initial framework;
2. Interview the students using the statistical reasoning assessment tool;
3. Analyze the students’ responses to the statistical reasoning assessment tool;
4. Refine the initial framework descriptors and revise the statistical reasoning assessment tool;
5. Interview the students a second time using the revised statistical reasoning assessment tool;
6. Scrutinize the statistical reasoning levels of the students for each construct in the refined framework;
7. Inspect the consistency of the students’ statistical reasoning levels across the four constructs; and
8. Distinguish the attributes of each statistical reasoning level.

### Participants

In the present study, ten tenth-grade students participated in task-based interviews. The number of participants used for framework validation in earlier studies [11,16,40–43] was between six and ten students. Among the participants, five were Chinese, three were Malay, and two were Indian. The students came from a secondary school in Johor, Malaysia, and all of them were sixteen years old.

Initially, the researcher sought consent from the Malaysian Ministry of Education and the Johor Education Department to conduct the research at the selected school. The researcher then obtained permission from the school headmaster and teachers for their students to participate in this study by showing them the approval letters from the Malaysian Ministry of Education and the Johor Education Department. The researcher asked the students to obtain written consent from their parents or guardians to participate in this study. Ten students were able to participate in this study; their approval letters and written informed consent letters were documented.

The participants were selected purposely to participate in this study because they already had prior knowledge about basic statistics. The topics that they had learned included the concept of class interval, the mode and mean of grouped data, cumulative frequency and measures of dispersion. The students had also been taught to present and interpret data in frequency polygons to solve problems and to use measures of central tendency and dispersion. However, these topics are to be covered within a month and are taught using conventional instructions based on the same textbooks in the school. Because of ethical issues, the privacy and anonymity of the participants are maintained in this study by assigning pseudonyms (S1 to S10) to each participant.

### Instrumentation

After the initial statistical reasoning framework was developed, the statistical reasoning assessment tool was constructed to refine and validate the framework. This assessment tool was designed based on the initial statistical reasoning framework to evaluate students’ statistical reasoning levels across the four constructs. The topics of descriptive statistics covered in this assessment tool were measures of central tendency and measures of variability. This assessment tool contained five tasks, with 56 items in total. Each item was associated with the sub-processes of four main constructs. The initial validation of this assessment tool, including content validity and inter-coder reliability, was performed in a previously published study [12]. The assessment tool was thus employed in the first task-based interview.

After the first task-based interview, the statistical reasoning assessment tool was revised because many items were found to be incapable of assessing the students’ statistical reasoning levels. The number of items was also reduced to 51 after the revision. The revised statistical reasoning assessment tool was employed in the second task-based interview. The amendment of this assessment tool is discussed in the findings section.

### Data Collection

In this study, data were collected via individual task-based interviews. As asserted by Goldin [44], task-based interviews play a crucial role as a research-based tool for assessing the subject matter. During the task-based interview, a statistical reasoning assessment tool was given to the students, who were then asked to work through the tasks using the computer when needed and to write down their answers on the answer sheets. The interviews were conducted either at school or at students’ homes, and the duration of the interview was two to three hours. The sessions were video-taped using a video camcorder, and the recordings were then transcribed, tabulated, and coded using NVivo 10 software.

### Data Analysis

The interview protocol was coded based on the descriptors of the refined statistical reasoning framework. Two raters evaluated the coding of the interview protocol for all students in five tasks. An example of coding for D2L5 (construct X Level) for the interview protocol is shown in Table 2. The raters had to select (√) if they agreed and (X) if they disagreed with this coding. The intercoder reliability was then computed according to their agreement. Cohen’s kappa was also calculated. Discussion continued until consensus was attained on dissimilar judgements on the coded responses. The same steps were replicated after the second task-based interview was conducted. The percentage of agreement was marked at 95.1%, which meant that the interview protocol was rationally reliable because it had passed the designated 70% threshold [45]. The value of Cohen’s kappa was 0.79, which was deemed good because it exceeded 0.7 [46].

**Table 2:** Table for interview protocol.

		Construct x Level	Agree (√) / Disagree (X)
The researcher:	Look at question no. 1; what does this graph tell you? Explain your answer.		
S3:	(looking at the question paper and thinking) This graph tells me the, umm, number of scores obtained by students on the statistics test.	D2L5	
The researcher:	How do you know? Can you show me?		
S3:	(looking at the paper, smiling) The title… aaah. The… the frequency is 30.		
The researcher:	How do you get 30?		
S3:	Aaah… (pointing to the histogram) Total number of frequency… in the graph… obtained, aah… by the score.		
The researcher:	Is there anything else you would like to add?		
S3:	This graph is negatively skewed.		
The researcher:	How do you know it is negatively skewed?		
S3:	Because the, aaah… it is skewed to the left. The left tail is longer.		
The researcher:	Anything else?		
S3:	(looking at her question paper) The highest frequency is 6, and the lowest frequency is 1. Eh… the highest frequency is 10, and the lowest is 1, 2, and 3.		
The researcher:	Can you show me?		
S3:	Aaah… 6 is the highest, and 1 is the lowest. (looking at the researcher)		

Statistical reasoning levels among the students were determined by calculating mean values for the codes. In Table 3, for describing data, Level 1 of D1 was multiplied by 2, Level 2 of D1 was multiplied by 3, Level 5 of D1 was multiplied by 3, Level 5 of D2 was multiplied by 1, Level 1 of D3 was multiplied by 1, and Level 2 of D3 was multiplied by 1. Subsequently, the researcher summed all the products and obtained a total sum of 31. This sum was then divided by the total number of items for describing data (11) to obtain the mean of 2.82, as shown in Table 3(1). For organizing and reducing data, Level 5 of O1 was multiplied by 3, Level 1 of O2 was multiplied by 1, Level 5 of O2 was multiplied by 2, Level 1 of O3 was multiplied by 2, and Level 2 of Q3 was multiplied by 1. The results of these products were summed to obtain a total of 30. Then, 30 was divided by 9 (the total number of items for organizing and reducing data) to obtain a mean of 3.33, as displayed in Table 3(2). For representing data, Level 5 of R1 was multiplied by 9, Level 2 of R2 was multiplied by 1, and Level 2 of R3 was multiplied by 1. Next, each product was summed to obtain 49, which was divided by 11 (the total number of items for representing data) to yield a mean of 4.45, as demonstrated in Table 3(3). For analyzing and interpreting data, Level 2 of A1 was multiplied by 3, Level 5 of A1 was multiplied by 1, Level 4 of A2 was multiplied by 2, Level 5 of A2 was multiplied by 1, Level 1 of A3 was multiplied by 3, Level 2 of A3 was multiplied by 1, Level 3 of A3 was multiplied by 1, Level 4 of A3 was multiplied by 5, and Level 5 of A3 was multiplied by 3. The total sum of the products (67) was divided by 20 (the total number of items for analyzing and interpreting data) to achieve a mean of 3.35, as indicated in Table 3(4).

**Table 3:** Frequency of codes assigned to S8’s responses.

Construct	Level	Level 1 Idiosyncratic	Level 2 Verbal	Level 3 Transitional	Level 4 Procedural	Level 5 Integrated Process
Describing Data		D1L1	D1L2	D1L3	D1L4	D1L5
		2	3			3
		D2L1	D2L2	D2L3	D2L4	D2L5
						1
		D3L1	D3L2	D3L3	D3L4	D3L5
		1	1			
Organizing and Reducing Data		O1L1	O1L2	O1L3	O1L4	O1L5
						3
		O2L1	O2L2	O2L3	O2L4	O2L5
		1				2
		O3L1	O3L2	O3L3	O3L4	O3L5
		2	1			
Representing Data		R1L1	R1L2	R1L3	R1L4	R1L5
						9
		R2L1	R2L2	R2L3	R2L4	R2L5
			1			
		R3L1	R3L2	R3L3	R3L4	R3L5
			1			
Analyzing and Interpreting Data		A1L1	A1L2	A1L3	A1L4	A1L5
			3			1
		A2L1	A2L2	A2L3	A2L4	A2L5
					2	1
		A3L1	A3L2	A3L3	A3L4	A3L5
		3	1	1	5	3

$$\text{Mean for describing data}=\frac{2\left(1\right)+3\left(2\right)+3\left(5\right)+1\left(5\right)+1\left(1\right)+1\left(2\right)}{11}=\frac{31}{11}=2.82\left(\text{Level }3\right)$$ (1)

$$\text{Mean for organizing and reducing data}=\frac{3\left(5\right)+1\left(1\right)+2\left(5\right)+2\left(1\right)+1\left(2\right)}{9}=\frac{30}{9}=3.33\left(\text{Level }3\right)$$ (2)

$$\text{Mean for representing data}=\frac{9\left(5\right)+1\left(2\right)+1\left(2\right)}{11}=\frac{49}{11}=4.45\left(\text{Level }4\right)$$ (3)

$$\text{Mean for analyzing and interpreting data}=\frac{3\left(2\right)+1\left(5\right)+2\left(4\right)+1\left(5\right)+3\left(1\right)+1\left(2\right)+1\left(3\right)+5\left(4\right)+3\left(5\right)}{20}=\frac{67}{20}=3.35\left(\text{Level }3\right)$$ (4)

A student was considered to have achieved Level 2 statistical reasoning if the mean was larger than 1.5 but less than or equal to 2.5. If the mean was larger than 2.5 but less than or equal to 3.5, a student was regarded as having Level 3 statistical reasoning, and so forth. For example, in Table 3, student S8 achieved a mean of 2.82 for describing data, which means that S8 was classified under Level 3. The mean value obtained by S8 for organizing and reducing data was 3.33: hence, she was at Level 3. Meanwhile, for representing data, her response was categorized as Level 4, with a mean of 4.45. Her response for analyzing and interpreting data was categorized as Level 3, with a mean of 3.35. After the statistical reasoning level of each student across the four constructs had been determined, the steadiness of their statistical reasoning levels was examined to ensure the consistency of the framework. Examples of each construct for the five levels are provided. All these data supported the validity of the refined statistical reasoning framework.

## Findings

### Refinements of the Framework

The students’ responses from the first and second task-based interviews were used to refine the descriptors of the initial statistical reasoning framework. Two major refinements were made. The first involved altering the existing descriptors to be equivalent to the student responses in the interviews. The second refinement involved inserting new phrases into the initial framework to reduce distinctions and discrepancies between the student responses and the initial framework. The refined statistical reasoning framework is shown in Table 4, which presents the modified descriptors in italic font and the removed descriptors in strikethrough.

**Table 4:** Refinements of the Statistical Reasoning Framework.

Construct	Level	Level 1 Idiosyncratic	Level 2 Verbal	Level 3 Transitional	Level 4 Procedural	Level 5 Integrated Process
Describing Data		D1L1	D1L2	D1L3	D1L4	D1L5
		Does not extract and generate *Reads* information from the data or graph *but is completely incorrect*	Extracts and generates *Reads* some information from the data or graph verbally but is ambiguous or unclear *incomplete or partially accurate*	Extracts and generates *Reads* one or two *more than one* dimension of information from the data or graph *correctly but is unable to relate it to the actual data or graph*	Extracts and generates *Reads* information from the data or graph correctly *but cannot fully provide explanation*	Extracts and generates *Reads* information from the data or graph completely *in a complete way and is able to integrate information correctly*
		D2L1	D2L2	D2L3	D2L4	D2L5
		Does not Shows awareness of the displayed attributes of graphical representation *but is completely incorrect*	Shows awareness of the displayed attributes of graphical representation orally but *is incomplete or partially* partly correct *accurate*	Shows little *some* awareness of the displayed attributes of graphical representation *correctly but is unable to relate to the actual data or graph*	Shows some awareness of the displayed attributes of graphical representation *correctly but cannot fully provide explanation*	Shows complete awareness of the displayed attributes of graphical representation *and is able to explain and relate to the actual data or graph*
		D3L1	D3L2	D3L3	D3L4	D3L5
		Does not Recognizes general features of the graphical representation *but is completely incorrect*	Recognizes general features of the graphical representation in words but *is incomplete or* partially accurate	Recognizes one or two *more than one* general features of the graphical representation *correctly but is unable to relate to the actual data or graph*	Recognizes general features of the graphical representation accurately *correctly but cannot fully provide explanation*	Recognizes general features of the graphical representation completely *in a complete way and is able to explain and relate to the actual data or graph*
Organizing and Reducing Data		O1L1	O1L2	O1L3	O1L4	O1L5
		Unable to organize the data into a computer system	Provides oral statements when organizing the data into a computer system but is only partly correct *incomplete or partially accurate*	Organizes the data into a computer system with major mistakes *correctly but is unable to relate to the actual data or graph*	Organizes the data into a computer system with minor mistakes *correctly but cannot fully provide explanation*	Organizes the data into a computer system in the right way *a complete way and is able to explain and relate to the actual data or graph*
		O2L1	O2L2	O2L3	O2L4	O2L5
		Unable to reduce the data using measures of center, either by calculation or aided by technology *central tendency*	Reduces the data using measures of center *central tendency* in words but only accurate to some extent *is incomplete or partially accurate*	Reduces the data using measures of center *central tendency* with major errors, either by calculation or aided by technology *correctly but is unable to relate to the actual data or graph*	Reduces the data using measures of center *central tendency* with minor errors, either by calculation or aided by technology *correctly but cannot fully provide explanation*	Reduces the data using measures of center central tendency completely, either by calculation or aided by technology *in a complete way and is able to explain and relate to the actual data or graph*
		O3L1	O3L2	O3L3	O3L4	O3L5
		Unable to reduce the data using measures of spread, either by calculation or aided by technology	Reduces the data using measures of spread orally either by calculation or aided by technology but only accurate to some extent *is incomplete or partially accurate*	Reduces the data using measures of spread with major faults, either by calculation or aided by technology *correctly but is unable to relate to the actual data or graph*	Reduces the data using measures of spread with minor faults, either by calculation or aided by technology *correctly but cannot fully provide explanation*	Reduces the data using measures of spread completely, either by calculation or aided by technology *in a complete way and is able to explain and relate to the actual data or graph*
Representing Data		R1L1	R1L2	R1L3	R1L4	R1L5
		Demonstrates data sets graphically using the computer without precise display	Provides verbal statements when demonstrating data sets graphically using the computer but only *is incomplete* or partially correct accurate	Demonstrates data sets graphically using the computer with major errors *correctly but is unable to relate to the actual data or graph*	Demonstrates data sets graphically using the computer with minor errors *correctly but cannot fully provide explanation*	Demonstrates data sets graphically using the computer with a valid display *in a complete way and is able to explain and relate to the actual data or graph*
		R2L1	R2L2	R2L3	R2L4	R2L5
		Does not Identifies different representations for the same data set *but is completely incorrect*	Identifies different representations for the same data set in words but only *is incomplete or* partially accurate	Identifies one or two *more than one* aspect of different representations for the same data set *correctly but is unable to relate to the actual data or graph*	Identifies different representations for the same data set in the correct way *correctly but cannot fully provide explanation*	Identifies different representations for the same data set in a complete way and comprehensive way *is able to explain and relate to the actual data or graph*
		R3L1	R3L2	R3L3	R3L4	R3L5
		Does not Judges the effectiveness of two different representations for the same data set *but is completely incorrect*	Judges the effectiveness of two different representations for the same data set orally but *is incomplete or* partially correct accurate	Judges one or two *more than one* effectiveness of two different representations for the same data set *correctly but is unable to relate to the actual data or graph*	Judges the effectiveness of two different representations for the same data set accurately *correctly but cannot fully provide explanation*	Judges the effectiveness of two different representations for the same data set completely *in a complete way and is able to explain and relate to the actual data or graph*
Analyzing and Interpreting Data		A1L1	A1L2	A1L3	A1L4	A1L5
		Does not Makes comparisons within the same data sets *but is completely incorrect*	Makes some comparisons within the same data sets verbally but is incomplete *or partially accurate*	Makes one or two *more than one* comparison within the same data sets *correctly but is unable to relate to the actual data or graph*	Makes comparisons within the same data sets correctly *but cannot fully provide explanation*	Makes comparisons within the same data sets completely *in a complete way and is able to explain and relate to the actual data or graph*
		A2L1	A2L2	A2L3	A2L4	A2L5
		Does not Makes comparisons between two different data sets *but is completely incorrect*	Makes comparisons between two different data sets in words but is somewhat incorrect *incomplete or partially accurate*	Makes one or two *more than one* comparison between two different data sets *correctly but is unable to relate to the actual data or graph*	Makes comparisons between two different data sets accurately *correctly but cannot fully provide explanation*	Makes comparisons between two different data sets completely *in a complete way and is able to explain and relate to the actual data or graph*
		A3L1	A3L2	A3L3	A3L4	A3L5
		Does not Makes a prediction, inference or conclusion from the data or graphs *but is completely incorrect*	Makes a prediction, inference or conclusion from the data or graphs in words but is incomplete *or partially accurate*	Makes one or two a prediction, inference or conclusion from the data or graphs *correctly but is unable to relate to the actual data or graph*	Makes a prediction, inference or conclusion from the data or graphs in the appropriate way *correctly but cannot fully provide explanation*	Makes a prediction, inference or conclusion from the data or graphs in a complete and comprehensive way *and is able to explain and relate to the actual data or graph*

The sub-processes of the framework were also revised. The sub-process of describing data was changed from ‘extracting and generating information from the data or graph’ to ‘reading information from the data or graph’. This change was made because the word ‘reading’ was deemed more suitable for direct problems, such as finding the highest and lowest values from the data or graph. With respect to the sub-process of organizing and reducing data, the phrase ‘either by calculation or aided by technology’ was eliminated because some questions were no longer part of the sub-processes of the constructs, as they were not useful for assessing students’ statistical reasoning. An example is the question that required students to record values from the computer. Moreover, the word ‘center’ confused the students while they were solving the tasks; thus, it was altered to ‘measures of central tendency’.

#### Refinements of describing data

The phrase ‘but completely inaccurate’ was added to the descriptors of D1, D2 and D3 to be consistent with the definition of idiosyncratic reasoning. This adjustment was made because the students had solved the tasks incorrectly. In addition, the phrase ‘ambiguous or unclear’ was changed to ‘incomplete or partially accurate’ at Level 2 of D1 because student responses were at times incomplete or only partially correct although they were able to attain precise information from the data or graph. Furthermore, some students were capable of gleaning more than a single piece of information from the data or graph. Thus, the descriptor of D1L3 was changed from ‘one or two dimensions’ to ‘more than one dimension’. Moreover, the phrase ‘are incomplete’ was added to D2L2 and D3L2. This change was made because students could not provide sufficient responses concerning the graph, even when they were probed by the researcher. Additionally, ‘some’ was used to replace ‘little’ in D2L3, and ‘some’ was erased in D2L4 to correspond to the definition of transitional and procedural reasoning.

The descriptor of D3L3, ‘one or two general features’, was modified to ‘more than one general feature’ because it was inappropriate for an item that asked students to portray the distribution of the graph with regard to its shape, as well as measures of central tendency and variability. To match with the definition of transitional reasoning, the phrase ‘correctly but is unable to relate to the actual data or graph’ was added at Level 3. In addition, to match the definition of procedural reasoning, the phrase ‘correctly but cannot fully provide an explanation’ was inserted into Level 4. At Level 5 of D1, the phrase ‘in a complete way and is able to integrate information correctly’ was added because the students were required to read the information completely and incorporate it accurately. Additionally, the phrase ‘and is able to explain and relate to the actual data or graph’ was included because the students had to achieve Levels 3 and 4 before reaching Level 5, which was related to interpreting the data or graph and giving justification.

#### Refinements of organizing and reducing data

Some items were no longer linked to any sub-processes of O2 or O3, but they were retained in the statistical reasoning assessment tool because those items (for example, the item that required students to mark the check box and record values from the computer) involved only procedural steps and could not elicit students’ statistical reasoning. Therefore, in each descriptor of O2 and O3, the phrase ‘either by calculation or aided by technology’ was removed. To match the definition of statistical reasoning levels, new phrases were inserted into other descriptors of organizing and reducing data.

#### Refinements of representing data

The descriptor of R2L3 was modified from ‘one or two aspects’ to ‘more than one aspect’ because some students were able to provide more than two aspects when solving the item asking them to explain how the box plot was associated with its corresponding histogram. Moreover, the phrase ‘judges one or two elements of effectiveness’ was changed to ‘judges more than one effectiveness’, as students were expected to give their opinions and reasoning comprehensively. In addition, other descriptors were edited to reflect the definition of statistical reasoning levels more closely.

#### Refinements of analyzing and interpreting data

For A1L3 and A2L3, the phrase ‘one or two comparisons’ was changed to ‘more than one comparison’ because the students were capable of making more than two comparisons between the same or different data sets. The phrase ‘makes one or two predictions, inferences or conclusions’ at Level 3 of A3 was also altered to ‘makes a prediction, inference or conclusion’. This change is made because the students generally gave only one prediction or conclusion for the task. Furthermore, other descriptors were revised by inserting new phrases to more closely reflect the definition of statistical reasoning levels.

### Amendment of Statistical Reasoning Assessment Tool

In the first interview, the researcher found that many items in the technology-based statistical reasoning assessment tool failed to assess students’ statistical reasoning ability. As a result, those items were again amended for use in the second interview. For describing data, the phrase ‘Explain your answer’ was added to each item, as displayed in Table 5. For instance, for the initial item ‘What are the highest and lowest amounts of protein (in grams) for various fast food sandwiches?’, the students were only able to mention the highest and lowest values; thus, their reasoning could not be traced. By adding the phrase ‘Explain your answer’, the students were given the opportunity to elaborate on how they had obtained the answer and more clearly demonstrate how they made sense of the statistical ideas.

**Table 5:** Examples of amended items for four constructs.

Constructs	Code	Sub-processes	Initial Items	Amended Items
Describing Data	D1	Reading information from the data or graph	What are the highest and lowest amounts of protein (in grams) for various fast food sandwiches?	What are the highest and lowest amounts of protein (in grams) for various fast food sandwiches? Explain your answer.
	D2	Showing awareness of the displayed attributes of graphical representation	What does this graph tell you?	What does this graph tell you? Explain your answer.
	D3	Recognizing general features of the graphical representation	Describe the distribution of the graph with respect to its shape, center and variability.	Describe the distribution of the graph with respect to its shape, measures of central tendency, and variability. Explain your answer.
Organizing and Reducing Data	O1	Organizing the data into a computer system	Organize the data into a GeoGebra spreadsheet.	Organize the data into a GeoGebra spreadsheet. Explain how.
	O2	Reducing the data using measures of central tendency	What is the meaning of the graph? Explain your answer.	What is the mean of the graph? Explain your answer.
			Record the values of the mean and median from the computer.	(Eliminated)
	O3	Reducing the data using the measures of spread	What is the range of the graph? Explain how you know.	What is the range of the graph? Explain your answer.
			Record the values of standard deviation from the computer.	(Eliminated)
Representing Data	R1	Demonstrating the data sets graphically using the computer	Draw the graph using a GeoGebra dynamic worksheet by dragging the red circle. Select the check box of Show histogram, Show mean and Show median.	Draw the graph using a GeoGebra dynamic worksheet by dragging the red circle. Select the check box of Show histogram, Show mean and Show median. Explain how you did so.
			Represent the data in another way.	Represent the data in another way. Explain your answer.
	R2	Identifying different representations for the same data set	Describe how the box plot is related to its matching histogram.	Describe how the box plot is related to its matching histogram. Explain your answer.
Analyzing and Interpreting Data	A1	Making comparisons within the same data set	Compare the results in question 15 with those in question 14. What do you observe? Explain why.	(Move to A2)
	A2	Making comparisons between two different data sets	Compare the distribution of both box plots with respect to shape, center and variability.	Compare the distribution of both box plots with respect to shape, measures of central tendency, and variability. Explain your answer.
	A3	Making a prediction, inference or conclusion from the data or graphs	Which measures of center are the most suitable to be used to represent the score obtained by students? Explain why.	Which measures of central tendency are the most suitable to be used to represent the score obtained by students? Explain why.
			Make a conclusion from the data on unemployment rates of males and females.	Draw a conclusion from the data on unemployment rates of males and females. Explain why.

For organizing and reducing data, variations of the phrase ‘Explain how you did so’ were also added to the item that asked the students to organize the data into the GeoGebra spreadsheet. The students were required to explain how they entered data into the computer step by step. The phrase ‘Explain your answer’ was also added to other items. For instance, in the question that involved finding the standard deviation, the students were required to perform some calculations to obtain the answer. They then had to explain how they had obtained the answer and what they understood about standard deviation. Furthermore, the items in O2 and O3 that asked the students to record values from the computer were eliminated because these items involved only stating practical steps that did not require statistical reasoning.

In Table 5, most items for representing data were altered by adding variations of the phrase ‘Explain how’. This change was made because the students were expected to clarify how they had created the graphical representations step by step. For example, when the students were asked to draw the frequency polygon, they had to construct it using a GeoGebra spreadsheet and then elucidate the steps to construct it. Some items for analyzing and interpreting data were amended as well, as displayed in Table 5. Initially, the item ‘Compare the results in question 15 with those in question 14. What do you observe? Explain why’ was wrongly included under A1 because the researcher thought that the results in questions 15 and 14 were the same data set. However, because they were actually two different data sets, the item was then moved to A2 after two students each obtaining a score of 1 were added to the graph. The phrase ‘Explain why’ was also added to several items because the students were expected to explicate their reasoning. For example, in the item ‘Draw a conclusion from the data on unemployment rates of males and females’, the students were expected to not only draw the conclusion but also explain their conclusion.

### Analysis of Statistical Reasoning Levels for Students across Four Constructs

**Fig 1:**
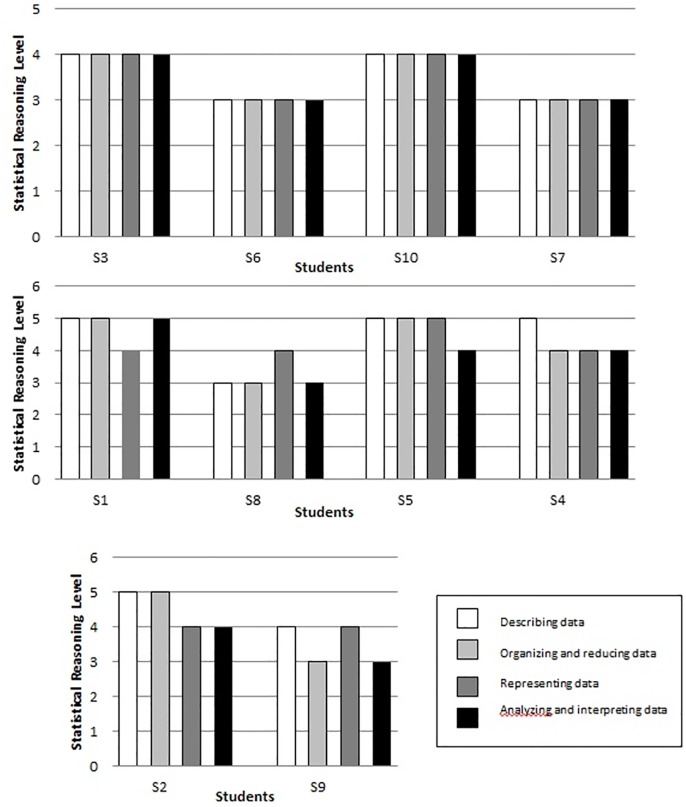
Students’ statistical reasoning levels across four constructs.

Fig 1 demonstrates the statistical reasoning levels among the students across the four constructs. The figure shows that four students (S3, S6, S7, and S10) had stable statistical reasoning levels for the four constructs. These students had the same capability in describing data, organizing and reducing data, representing data, and analyzing and interpreting data. Furthermore, we found that four students (i.e., S1, S8, S5, and S4) had the same statistical reasoning levels for three constructs. One of the constructs was lower or higher than the other three. For instance, student S1 appeared to be at Level 5 in describing data, organizing and reducing data, and analyzing and interpreting data but was at Level 4 in representing data. Moreover, two students possessed the same statistical reasoning levels for two constructs, namely, S2 and S9. These students achieved Level 4 in describing data and representing data and Level 3 in organizing and reducing data as well as in analyzing and interpreting data. This result indicates that these two students had inconsistent statistical reasoning levels across the four constructs.

In general, the students’ statistical reasoning levels indicated strong internal consistency across the four constructs, as eight of the ten students achieved consistency in the four constructs. Thus, 80% of the students had reached the same levels for at least three of the four constructs. As claimed by Jones et al. [11], improving the cohesion of the framework crucially involves ensuring stable and consistent statistical reasoning levels of the students.

### Analysis of Statistical Reasoning at Each Level

The statistical reasoning levels of the students across the four constructs were analyzed based on the refined statistical reasoning framework. Examples of students’ responses in five levels are discussed below.

#### Level 1 summary

As illustrated in Fig 1, no student was categorized as Level 1, idiosyncratic reasoning, but some responses from the students were at this level. As previously hypothesized, the characteristics of students at Level 1 are similar to the elements of the prestructural level of the SOLO model in the ikonic mode.

With respect to describing data, the evidence showed that the students could read the information from the data or graph, but their solution was wrong. They also displayed awareness of the graphical characteristics presented and were able to recognize the common features in the graphs, although they did so incorrectly. For instance, when student S8 was attempting to solve a question in Task 5 that involved finding the highest and lowest number of weeks needed to finish reading a storybook, she was not aware that the x-axis signified the number of weeks; she referred to the y-axis instead, as revealed in the following excerpt from her interview protocol. This error was also found in the study by Cooper and Shore [27].

The researcher: Look at the question: The following graphs illustrate the number of weeks used by the students from class 4A and 4B to finish reading a storybook. Question no. 1. What are the highest and lowest number of weeks used by the students from class 4A to finish reading a storybook? Explain your answer.

S8: (Looking at her paper) The highest number of week is 6, and the lowest number is 2.

The researcher: Can you show me how you got the answer?

S8: (Pointing to the histogram on the paper) it…this is the highest number, and this is the lowest number.

**Fig 2:**
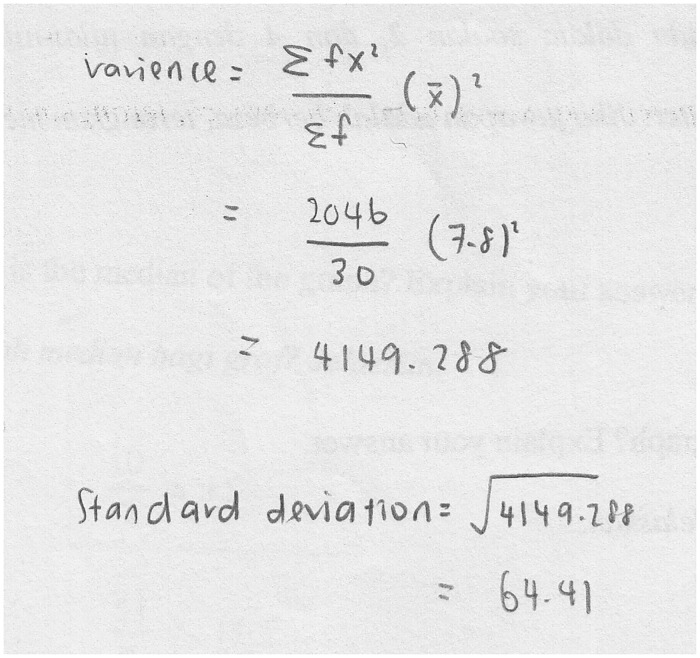
S8’s solution to find the standard deviation of the graph.

In relation to organizing and reducing data, the students were incapable of organizing the data into the GeoGebra spreadsheet, and they inaccurately reduced the data using measures of central tendency and variability. For example, S8 attempted to calculate the standard deviation of the graph in Task 1, but the wrong formula was used because she missed a minus sign, as displayed in Fig 2. Such mistakes corroborated the findings by Kourkoulus and Tzanakis [47], who discovered that their students did not understand standard deviation, as they were unable to calculate standard deviation when the formula was not given.

**Fig 3:**
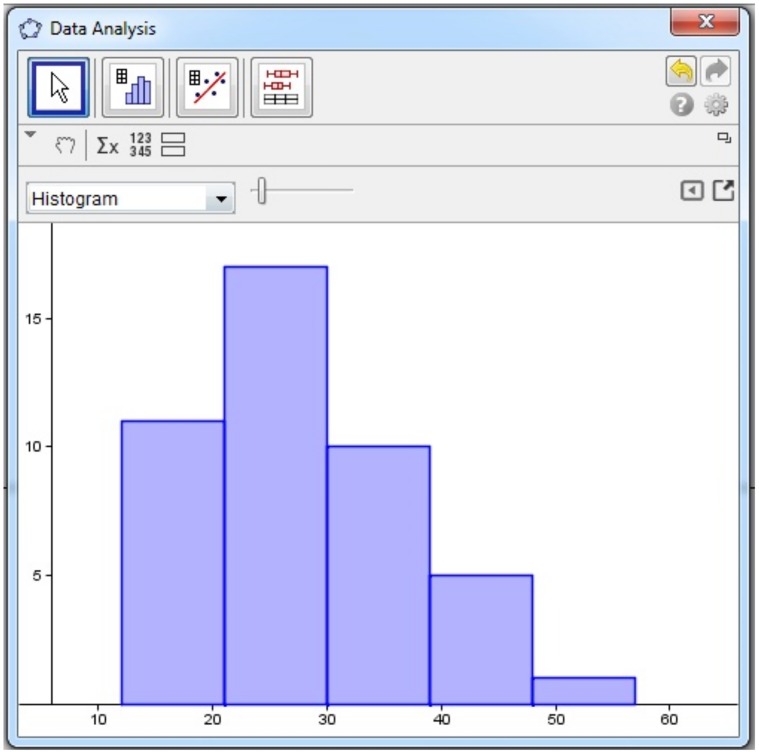
S4’s construction of a frequency polygon.

For representing data, the students failed to draw proper graphs using a computer. Additionally, they were unable to recognize different representations of the same data set. The students were also incapable of evaluating the efficiency of two different demonstrations for the same data set. For instance, the frequency polygon was wrongly illustrated by student S4 in Task 2 because he could not differentiate between the histogram and the frequency polygon, as illustrated in Fig 3.

With regard to analyzing and interpreting data, the students could not accurately make comparisons within the same data set and between two different data sets. Moreover, they failed to draw correct conclusions, predictions or inferences from the data or graph. To illustrate this point, student S5 made an incorrect prediction for Task 5 because she perceived that the histogram with a flat and smooth distribution had a smaller standard deviation than the histogram with bars of dissimilar height, as shown in the following interview protocol. This type of mistake was also reported by Garfield, delMas, and Chance [48].

The researcher: Next. Umm… The teacher did a survey of the number of weeks used by the students from class 4A and 4B to finish reading a book during the school holidays. The following data indicated the results of the survey. Question no. 7, Predict which class has the larger standard deviation. Explain why.

S5: Class 4B because…

The researcher: Why?

S5: I think because class 4B will be more spread out than class 4A.

The researcher: Can you show me how you know it is more spread out?

S5: (Pointing to the table on the paper) Because when we see the data, class A is just, aaah… the values are the same. So the graph will be like… straight away (Drawing a straight line) the histogram. But for 4B, it increases, it decreases (moving her hand), the graph will be more spread out from the mean. This is the mean… aaah, for this, the mean is 3, median 3, so it is less spread out. The difference also… interquartile range… ehem… OK.

#### Level 2 summary

Similar to the results for Level 1, idiosyncratic reasoning, no student was categorized at Level 2, verbal reasoning. Nevertheless, reasoning at this level was shown in some of the students’ responses. As noted earlier, this level is consistent with the unistructural level of the SOLO model at the concrete symbolic mode.

In describing data, students at this level could read some of the information from the data or graph verbally, but it was incomplete or only partly accurate. In addition, they exhibited incomplete or partly accurate awareness of the displayed features of graphs. The students could recognize general aspects of the graphs, but their responses were incomplete or only partially accurate. In the interview protocol below, for example, student S8 was required to examine the general attributes of a frequency polygon in terms of shape, measures of central tendency and variability in Task 2. She was able to identify the shape correctly (i.e., skewed to the right). However, she could not explain the reason for this shape: she perceived that most of the data were on the right side if it was skewed to the right. This misconception was also observed by Lee, Zeleke and Wachtel [49]. For the measure of central tendency, she asserted that the correct answer concerned the mean. However, upon prompting, she altered her response to the median, and she was able to give the correct response: the mean was larger than the median. For the measure of variability, she attempted to use the range, but this response was incorrect because the highest amount was computed incorrectly in the earlier question.

The researcher: Question no. 4, Describe the distribution of the graph in terms of its shape, center and variability. Explain your answer.

S8: (Looking at the screen and her paper) The graph shows… the graph is skewed to… right.

The researcher: Uh… How do you know it is skewed to the right? Can you explain?

S8: (Looking at the graph) More concentrated… at right…

The researcher: Can you show me? Where… where is it more concentrated on … on the right side? Can you show me? Which one?

S8: (Moving the cursor to the graph on the screen) Is it here? Eh, no… (touching her head and pointing her finger at her paper)

The researcher: Box plot? (laughing)

S8: (laughing)

The researcher: You want to choose the box plot, huh?

S8: Ha… (changing the graph on the screen)

The researcher: If you want to… you can…

S8: (Looking at the screen and her paper) Skewed to the right. The graph is skewed to right.

The researcher: Hm… can you explain?

S8: (Thinking) The right has… right has spread out… more, more at the right.

The researcher: Then how about… how about the center?

S8: The center is… median… median… (looking at the screen) the center is mean 27.625.

The researcher: Can you explain your answer? Show me… show me… the mean.

S8: (Looking at the screen) Median, ha… (laughing) center is median.

The researcher: So what is the median?

S8: 26.5…

The researcher: How about the variability?

S8: The median… the mean is larger than the median… skewed to the right, and the range… the range is 32.

The researcher: Ha… Can you explain how you got the 22, ha?

S8: (Looking at her paper) 32…

The researcher: 32? How do you get it?

S8: By using the larger number minus the lower number (pointing her finger to number 44 and 12)

**Fig 4:**
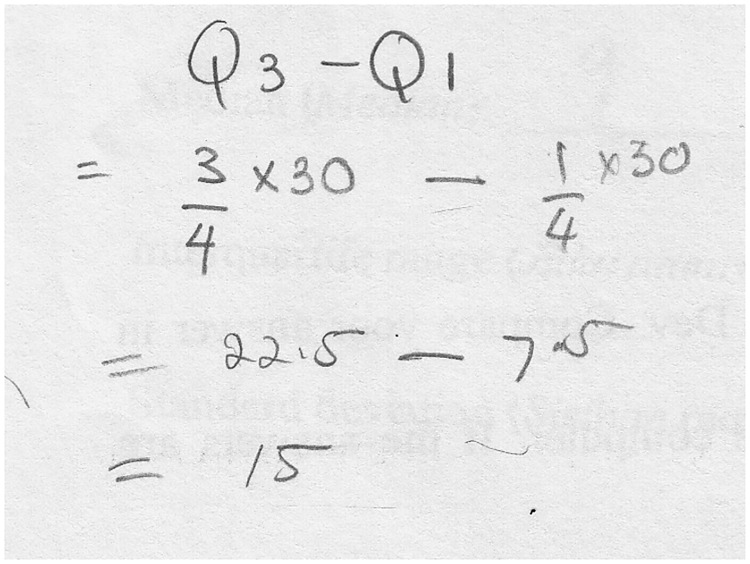
S7’s solution to find the interquartile range of the graph.

With respect to organizing and reducing data, the students managed to describe the process of organizing data using the computer, but their answers were incomplete or only partially correct. They reduced the data using measures of central tendency and variability, but their statements were also fragmented and somewhat inaccurate. For instance, student S7 could compute the interquartile range in Task 1, but she missed the steps for obtaining the 7.5th observation and the 22.5th observation from the histogram, as displayed in Fig 4.

As with representing data, the students could explicate the procedure for drawing the graph using the computer, but their answers were incomplete and only partially accurate. Additionally, they could distinguish the different representations and explain the effectiveness of the two representations for the same data set, but their answers were incomplete or only partially correct. For example, student S5 selected the histogram as a better representation when she was asked to choose between the histogram and the box plot in Task 2. Nonetheless, even after probing by the researcher, this student could give only one rationale—which was that the histogram could closely depict the shape of the distribution—as the following interview protocol illustrates.

The researcher: Aah… Question no. 8, Which graph do you think represents the data better, the histogram or the box plot? Explain why.

S5: I think the histogram. The histogram shows the shape of the distribution better.

The researcher: Any other reason?

S5: No (shaking her head), that’s all.

For analyzing and interpreting data, the students made incomplete or partially correct comparisons within the same data set and between the different data sets. They also drew incomplete or partly correct inferences, conclusions or predictions from the data or graph. For instance, when a question in Task 5 asked, ‘Are there any similarities or differences between the two graphs produced on the computer? Explain why’, student S9 demonstrated proficiency in stating that the similarities were in the interquartile range and the standard deviation, while the differences were in their mean and median. However, she did not provide any reason for this answer, as presented in the following excerpt from her interview protocol.

The researcher: Umm… Question no. 14, are there any similarities or differences between the two graphs produced on the computer? Explain why.

S9: The interquartile range and the standard deviation in both graphs are the same, and the mean and median of the two graphs are different.

The researcher: Why are the interquartile range and the standard deviation the same?

S9: (Examining the paper and shaking her head)

The researcher: How about the mean and median? Why are they different?

S9: (Examining the paper) They’re influenced by the, aah… the… (shaking head)

The researcher: Is there anything else you might like to add?

S9: (Shaking her head again)

#### Level 3 summary

A number of students were categorized as Level 3, transitional reasoning, for certain constructs. This level was concordant with the multi-structural level of the SOLO model at the concrete symbolic mode, as discussed above. None of the responses for the constructs of describing data and organizing and reducing data could be categorized at this level.

With regard to representing data, the students were able to construct the graphs properly using the computer, but they could not relate them to the actual data. The students could distinguish more than one feature of different depictions for the same data set and evaluate the effectiveness of two depictions for the same data set, but they could not relate them to the actual data or graph. For example, as shown in the following interview protocol, student S9 was capable of drawing the stem and leaf plot and providing a step-by-step explanation in Task 4, but she failed to relate the plot to the actual data.

The researcher: OK. Question no. 4. Construct a stem and leaf plot for each set of data. Explain how you did so.

S9: (Holding and clicking the mouse) Highlight the data and click the one variable for analysis and choose the stem and leaf plot.

The researcher: How are the data and the stem and leaf plot related to each other? (pointing to the screen)

S9: (Examining the screen and then shaking her head)

With reference to analyzing and interpreting data, the students were incapable of relating their answers to the actual data or graph when making comparisons within the same data set and between different data sets. The same problem occurred when they drew conclusions, predictions, or inferences from the data or graph. For example, when student S3 was asked to draw a conclusion from the data on instant noodle consumption for Taiwan and Malaysia in Task 3, he was able to solve the question, but he could not relate his answers to the actual data or graph. In this case, he provided only his own opinion—that Malaysians like instant noodles more than Taiwanese people—as demonstrated in the excerpt from his interview protocol.

The researcher: Question no. 8, Draw a conclusion from the data on instant noodle consumption for Malaysia and Taiwan. Explain why.

S3: (looking at his paper and thinking) Malaysia… instant noodle consumption for Malaysia is higher than Taiwan.

The researcher: Why is it higher? Explain why?

S3: Emm (thinking) because it is needed for…

The researcher: It is?

S3: Needed. For… food (smiling) (look at his paper)

The researcher: Any other reason?

S3: Mmm (looking at his paper and thinking) the Malaysian likes to eat instant noodle more than Taiwanese (smiling)

The researcher: Any other reason?

S3: Mmm (looking at his paper) no.

#### Level 4 summary

Several students were categorized under Level 4, procedural reasoning. As stated previously, this level was consistent with the relational level of the SOLO model in the concrete symbolic mode.

With regard to describing data, the students could read information from the data or graph precisely, but they could not provide a full explanation. Furthermore, they could not give complete justification, although they could consciously identify the displayed aspects of the graph and accurately identify its general traits. For instance, as indicated in the following interview protocol, student S2 managed to provide accurate responses for three traits when he was asked to describe the distribution of the histogram with respect to shape, measures of central tendency and variability in Task 1. His responses were as follows: (i) the graph was unimodal and negatively skewed, (ii) the mean was smaller than the mode and the median, and (iii) the range of the graph was from 1 to 11. Nonetheless, he misinterpreted the negatively skewed histogram when he explained the shape of the histogram.

The researcher: Question no. 11. Describe the distribution of the graph with respect to its shape, center and variability. Explain your answer.

S2: Shape… (Looking at the graph on his paper) the graph is unimodal and negatively skewed to the left… and the center…

The researcher: Can you explain first how you know it is skewed to the left and unimodal?

S2: It only has the higher frequency at one place (pointing to the histogram on the paper) and unimodal (scratching his head) and the graph is… and the graph is more compact on the right side, so it is negatively skewed.

The researcher: More compact?

S2: Ha… more compact on the right side, so it is negatively skewed.

The researcher: Then how about the center?

S2: The mean is lower than the median and the mode.

The researcher: Why do you say that?

S2: According to the negative distribution graph, the mean will always be lower than the median and the mode. This is proven by the …this calculator [pause] value.

The researcher: How about the variability?

S2: The variability… (Looking at his paper) the graph ranges from 1 to 11.

The researcher: Can you tell me more about that? Why do you say 1 to 11? Can you show me?

S2: Because the range is from 1 to 11 (Pointing on his paper) the lowest to the highest.

In organizing and reducing data, the students correctly organized the data on the computer, but they did not provide adequate explanations for their responses. Moreover, they failed to provide justification, although they had successfully reduced the data using measures of central tendency and variability. For instance, when a question for Task 1 asked, ‘What is the range of the graph? Explain your answer’, student S9 was able to obtain the range from the histogram and explain how she arrived at the answer (i.e., subtracting 1 from 11). However, she could not describe the meaning of the range, as displayed in this excerpt from her interview protocol.

The researcher: OK, now we look at question no. 7. What is the range of the graph? Explain your answer.

S9: (Looking at the laptop’s screen) The range is 10.

The researcher: Can you explain how you got 10?

S9: Eleven… (shaking head) Eleven minus one.

The researcher: Aah… can you show me how you got it?

S9: (Pointing at the screen) This 11… minus 1.

The researcher: So, aah… What do you mean by range?

S9: (Thinking and then shaking her head)

For representing data, the students were able to use the computer to display the data sets graphically but were unable to explain them properly. In addition, they were capable of identifying different graphs for the same data set and judging the usefulness of two different graphs for the same data set, but they failed to fully explain them. For example, when she was asked in Task 2 to describe how the box plot could be related to its matching histogram, student S10 was able to identify that the interquartile range and median of the box plot were comparable to the histogram. She also noted that the shapes of both graphs were skewed to the right and that the mean was greater than the median. Nevertheless, she gave the wrong reason for the rightward skewness, as she noted that a positive outlier would pull more data below the median, as revealed in the following interview protocol.

The researcher: Now, we proceed to question no, 7. Describe how the box plot is related to its matching histogram. Explain your answer.

S10: (thinking) the vertical lines in the box plot shows the mean… shows the median of the graph and the, umm… and the length from (pointing on screen), from here until here shows the… which represents the interquartile range.

The researcher: Here until here?

S10: Umm… (nodding head) the… from here until here (shows on the box line) shows the interquartile range… (nodding head) which is the difference from here… aaah, the difference between the third quartile and the first quartile. (nodding head)

The researcher: So is it the same for the histogram?

S10: (looking at screen) yes. (nodding head)

The researcher: How about the median? Just now you said that this one (shows on the screen) indicates the median, so the median is for the box plot or the histogram?

S10: The median… (thinking) emm (pointing to the screen), the median for the box plot and the histogram are same… which represents the… center of \ the graph. Mm.

The researcher: Is there anything else you would like to add?

S10: (thinking) The positive outlier… it pulls… ah, it is a positively skewed distribution because it has, aah… it has a positive outlier. Mm (nodding head)

The researcher: Is there anything else you would like to add?

S10: No. (shaking head)

The researcher: How about the shape? For both graphs?

S10: (thinking and looking at the screen) the shape of the graph is, aaah… emm… positively skewed or skewed to the right… and the mean is probably larger than the median. (nodding head)

The researcher: Can you show me how you… how you know it is skewed to the right?

S10: Umm. (Pointing to the screen) Because the positive outlier pulls more data below the median. Umm…

The researcher: Is there anything else you would like to add?

S10: (looking at her paper) no. (shaking her head)

With respect to analyzing and interpreting data, the students were proficient in making comparisons within the same data set and between the different data sets, but they could not provide full explanations. Additionally, they managed to make predictions, conclusions, or inferences from the data or graph, but they did not provide adequate justification for their responses. For example, as exemplified in the interview protocol below, when student S4 was asked to compare two different data sets, he replied that the interquartile range and the median remained the same, but the standard deviation and the mean had changed. However, he did not correctly justify the changes.

The researcher: OK. Question no. 13. Compare the results in Question 13 with Question 12. What can you observe? Explain why.

S10: (Referring to the answer on the paper) The mean and standard deviation have changed. But the median and interquartile range remained the same.

The researcher: Why?

S10: Because the data have changed.

The researcher: Why are the median and interquartile range still the same? And why did the mean and standard deviation change?

S10: I don’t know.

The researcher: Why is there a change after you added the two students?

S10: Because the number of students has changed.

#### Level 5 summary

A few students reached Level 5, integrated process reasoning. This level was consistent with the extended abstract level of the SOLO model in the formal mode, as described earlier.

In describing data, the students were able to read the information from the data or graph and incorporate this information accurately. In addition, they demonstrated complete awareness of the graph features displayed and were able to identify the common graphical attributes, explain them, and relate them to the actual data or graph. For instance, when the question in Task 1 asked, ‘What does this graph tell you? Explain your answer’, student S8 was aware of the graphical conventions, including the title of the histogram and its axis labels. Additionally, she managed to state that the values of the highest and lowest frequencies were 6 and 0, respectively. The number of students who scored between 1 and 11 was also correctly stated. All the frequencies in the histogram were summed to obtain the total frequency. In the following interview protocol, note that student S8 was able to state her reasoning by showing how she made sense of the graph when prompted by the researcher.

The researcher: Now we look at the question no. 1. What does this graph tell you? Explain your answer.

S8: (looking at the question paper) Umm… The graph shows the… the score obtained by students on the statistics test.

The researcher: How do you know? Can you show me?

S8: Show, aah? (Smiling)

The researcher: This one?

S8: This one… aaah, this one (pointing to the y-axis and x-axis) the highest frequency of the score is 6. (Laughing) There is one student who scored one, one student who scored two, one student who scored three, two students who scored four, and there are no students who scored five, three students who scored six, three students who scored seven, four students who scored eight, five students who scored nine, six students who scored ten and four students who scored eleven. And the highest fre—the lowest frequency is 0.

The researcher: Ha… How do you know?

S8: (Pointing her finger at the histogram) Score 5 is here.

The researcher: How about the highest?

S8: The highest frequency…the highest frequency is 6 (pointing to the 10 score).

The researcher: Aaah… Is there anything else you would like to add?

S8: (Shaking her head) No…

The researcher: How about the total frequency?

S8: Oh… (Looking at her question paper and calculating) the total frequency is 30.

The researcher: How do you know?

S8: Umm… (Pointing to the histogram) by adding all the frequency numbers in the graph.

**Fig 5:**
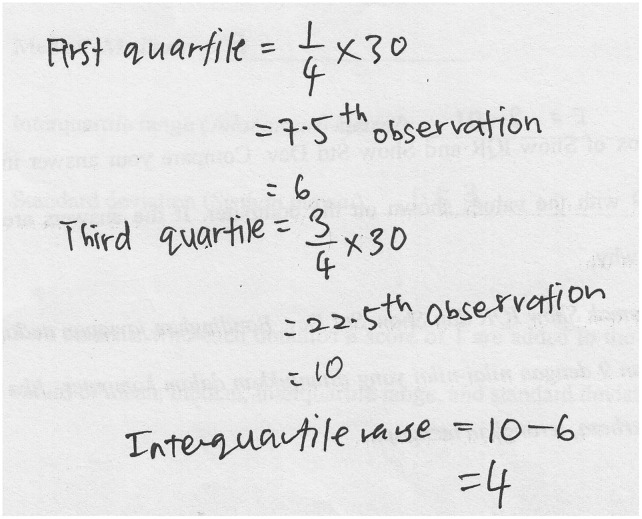
S5’s solution to find the interquartile range of the graph.

In terms of organizing and reducing data, the students were able to organize data using the computer and explain the relationships between the real data or graph and the reduced, organized data. Furthermore, they could also reduce the data using measures of central tendency and variability as well as relate these measures to the actual data or graph. For example, in Task 1, student S5 could compute the interquartile range accurately and explain the procedure used to obtain the answer, as illustrated in Fig 5. She also stated that the interquartile range was the difference between the third and first quartiles.

**Fig 6:**
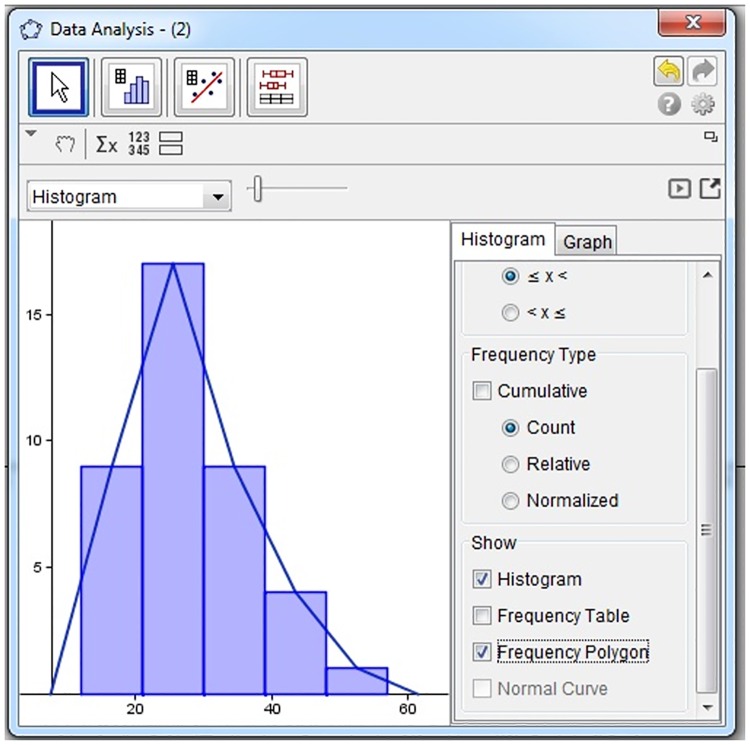
S3’s construction of a frequency polygon.

In representing data, the students were able to use the computer to represent data sets in a comprehensive way and to explain and relate to the actual data or graph. Moreover, they were able to identify different representations for representing the same data set and were competent in providing explanations and simultaneously relating them to the actual data or graph. The students were also able to evaluate the effectiveness of two different graphs for the same data set as well as to explain the relationships between the graphs and data. For instance, student S3 was able to draw the frequency polygon using a GeoGebra spreadsheet and to clarify the steps used to construct it in Task 2, as presented in Fig 6. He also explained that the reason for transforming the data into a frequency polygon was to clearly see the shape of the data.

In analyzing and interpreting data, the students made comprehensive comparisons within the same data set and between different data sets and were able to explain their relationships with the actual data or graph. The students were also able to justify their responses when making inferences, predictions and conclusions. For example, as shown in the following interview protocol, when the question required student S10 to compare the distribution of two box plots with reference to their shape, measures of central tendency, and variability in Task 3, she solved this question proficiently. For shape, she stated that the box plot for Malaysia was larger than that for Taiwan. For measures of central tendency, she claimed that the mean was larger than the median because the right tail was longer than the left tail, and she noted that the box had been shifted to the left side. For measures of variability, because the range was larger, she said that the variability of the Malaysian data was greater than that of the Taiwanese data.

The researcher: Question no. 7: Compare the distribution of both box plots with respect to shape, center and variability. Explain your answer.

S10: Aah, the box plot of… noodle consumption in Malaysia is wider than that in Taiwan.

The researcher: How do you know it is wider? Can you show me?

S10: (Pointing to the screen) Aah, because the length of the box plot is larger… of… Malaysia is larger than Taiwan.

The researcher: How about the center?

S10: Center… (looking at her paper and screen) emm. For Malaysian noodle consumption, the middle, the mean is larger than the median because, aah… the box… the box plot is shifted to the left side and the right tail is longer than the left tail.

The researcher: How about, aaah, Taiwan?

S10: (Looking at the screen) Umm, for Taiwan, umm… the… ah, the right tail is also longer than the left tail. This means the mean is larger than the median.

The researcher: Aah, what about the variability?

S10: Variability. The… the noodle consumption… distribution for Malaysian noodle consumption is more spread out that the Taiwanese noodle consumption because the range of… the range for Malaysia is larger than for Taiwan.

The researcher: What is the range for Malaysia?

S10: The range for Malaysia (looking at her paper), ah, is calculated by… (pointing to the screen) ah, calculating the difference from the max… by using the maximum minus the minimum. For Malaysia, the range is 4.4, while for Taiwan, the range is 1.5.

## Discussions

In this study, a framework for assessing students’ statistical reasoning levels across four constructs was formulated and validated. This framework provides a guideline for instructors to plan learning goals and design instruction and assessments. In addition, the assessment tool based on the statistical reasoning framework allows instructors to assess students’ statistical reasoning levels. Researchers can use this statistical reasoning assessment tool in future studies with students of different genders, grade levels, cultures, countries, and so forth. The processes of framework validation in the present study were adopted from previous studies including those of Jones, Thornton and Putt [40], Jones et al. [41], Jones et al. [42], Tarr and Jones [43], Jones et al. [11], and Mooney [16]. However, the validation processes in the present study were distinct from those in previous studies because the task-based interview was conducted twice and the statistical reasoning assessment tool was revised after the first task-based interview. The descriptors in the statistical reasoning framework were refined according to the students’ responses in the statistical reasoning assessment tool, leading to a better representation of students’ statistical reasoning levels. This step makes the framework more applicable and legitimate for future use by instructors. The amended statistical reasoning assessment tool was also more accurate for assessing students’ statistical reasoning. This validation process can be followed by other researchers seeking to validate their frameworks.

Stability in students’ statistical reasoning levels across the four constructs was sought in validating and formulating the framework. The statistical reasoning levels among the students were considered to be consistent because 80% of the students had achieved proficiency for at least three out of four constructs. Two previous studies have employed Garfield’s [10] model, i.e., Aquilonius [50] and Silva and Coutinho [51]. Both studies indicated that no students could accomplish Level 5 reasoning—integrated process reasoning. In contrast, some students in the present study were able to attain Level 5 proficiency. Nevertheless, only 40% of the students obtained the same levels in all four constructs. Therefore, some irregularities remain in the statistical reasoning framework. Therefore, further investigations should be conducted to improve and revise the statistical reasoning framework because consistency among students’ statistical reasoning levels is crucial for a coherent framework [11].

Examination of students’ statistical reasoning at each level and across the four constructs is also imperative to validate the framework. The findings indicated that the five levels of statistical reasoning [10] corresponded to the five levels of understanding of the SOLO model [14,18] as expected, i.e., idiosyncratic reasoning with the prestructural level, verbal reasoning with the unistructural level, transitional reasoning with the multistructural level, procedural reasoning with the relational level, and integrated process reasoning with the extended abstract level. At Level 1, although students attempted to answer the questions, their solutions were irrelevant and wrong because they solved the problems according to their own intuition [52]. At Level 2, the students had one relevant idea, but their answer was always incomplete or partly correct, although they had begun to use some statistical terms [52]. Furthermore, at Level 3, students had several relevant ideas but could not combine them together [18] and relate them to the data or graph. At Level 4, students could integrate the relevant ideas into a coherent whole. However, they still did not fully understand the statistical concepts [10] and were thus unable to provide a complete explanation. At Level 5, the students completely understood the statistical concepts and made generalizations and conceptualizations of the earlier integrated whole at a higher level. They were also able to apply abstract statistical ideas at this level [17]. In this study, one cycle of unistructural-multistructural-relational (UMR) knowledge within the concrete-symbolic mode was discovered. This finding was consistent with the results from the studies of Jones et al. [11] and Mooney [16].

Some misconceptions in the four constructs were also found in students’ responses to the statistical reasoning assessment tool, particularly at Level 1 (idiosyncratic reasoning) and Level 2 (verbal reasoning). Thus, students continued to encounter difficulties in describing data, organizing and reducing data, representing data, and analyzing and interpreting data. Thus, more instructions such as the Statistical Reasoning Learning Environment (SRLE) could potentially be used to reduce students’ misconceptions related to those constructs. As shown in Fig 1, only one student achieved Level 5, integrated process reasoning, in terms of representing, analyzing, and interpreting data. This finding indicates the difficulty that students might have in attaining Level 5 reasoning. Hence, these two constructs should be given particular emphasis in future studies.

## Conclusion

A framework for assessing statistical reasoning levels among high school students was developed and refined in this study. Statistical reasoning is an integral part of statistics education, and the formulation of the statistical reasoning framework in this study is an important foundation for teaching practice and assessment. The framework can be used to assess students’ statistical reasoning and determine their proficiency levels relative to the four listed constructs. Future studies should investigate the generalizability of the utility of the framework for examining statistical reasoning skills among students from different grade levels, genders, and cultural backgrounds. Further research is needed to improve this framework, to develop other statistical reasoning assessment tools, and to conduct novel instruction to better promote and evaluate students’ statistical reasoning ability.
